# Use of simulation in the evaluation of airway clearance devices

**DOI:** 10.1016/j.jped.2025.101435

**Published:** 2025-08-11

**Authors:** Maria Lucia S. Hristonof, Marina C. Amantéa, Fernando J. Lazzaretti, Marina M. Bernardes, Luiza F. Xavier, Sergio Luis Amantéa

**Affiliations:** a Pontifícia Universidade Catolica do Rio Grande do Sul (PUCRS), Faculdade de Medicina, Porto Alegre, RS, Brazil; b Pontifícia Universidade Católica do Rio Grande do Sul (PUCRS), Grupo de Pesquisa do Centro Infant, Porto Alegre, RS, Brazil

We sincerely thank the authors of the Letter to the Editor for their insightful comments, which highlight important limitations inherent to simulation-based studies, especially when evaluating interventions for foreign body airway obstruction (FBAO).

We fully recognize that mannequins do not reliably reproduce the anatomical and biomechanical characteristics of the human airway, nor the variability of real clinical choking situations. These limitations are legitimate and deserve to be discussed before any judgment is made.

However, we respectfully argue that experimental studies using high-fidelity mannequins offer relevant contributions, particularly in contexts where clinical trials in humans would be ethically unfeasible. This is especially relevant for FBAO, an acute and unpredictable condition in which patient safety precludes the design of randomized trials. As pointed out by Dunne et al.^1^ and Patterson et al.,^2^ simulation models allow for controlled, reproducible, and safe comparisons between different approaches, including suction devices and traditional maneuvers such as the Heimlich.

Our study did not aim to perfectly recreate a real-life scenario but to objectively quantify pressure parameters and success rates under standardized conditions. These data, although derived from a simulation model provide an ethical and reproducible method for analyzing the biomechanical effects involved, as also suggested by Juliano et al.^3^ We believe this type of experimental evidence can complement the clinical case reports available in the literature and contribute quantifiable information to support policy development and clinical guidance.^4^

Furthermore, international resuscitation councils and systematic reviews have emphasized the urgent need for further evaluations of suction devices, specifically recommending bench and simulation-based studies.^1^^,^^5^ ILCOR has still positioned itself against the routine use of suction-based airway clearance devices (weak recommendation, very low-certainty evidence).^6^ While limited, these studies are fundamental to inform future observational research and evidence-based regulatory decisions.

Our results point to an effective performance of both maneuvers, considering the outcome of the mannequin's airway clearance and patency. This finding motivates us to continue researching this and other suction devices that can bring benefits to the management of this medical emergency.

It is also important to clarify that our group has no commercial or institutional conflicts of interest related to any devices or companies associated with the topic. Our sole commitment is to the production of rigorous and independent scientific evidence that can support clinical and policy decisions based on data.

We would also like to address the relevant aspect raised in the discussion: the anatomical differences between adult and pediatric airways. Indeed, the pediatric airway presents unique features — such as smaller caliber, greater tracheal wall compliance, and a higher laryngeal position — which increase the risk of FBAO and may influence the application of certain maneuvers. However, for biomechanical comparative analysis in simulated models (e.g., pressure measurement or object displacement), these differences tend to have a secondary impact. The choice of a standardized adult mannequin model follows the methodology adopted in other studies^1^^,^^2^ and aims to ensure reproducibility and variable control, allowing direct comparison across techniques.

In addition, we believe it is essential to highlight that the construction of robust scientific evidence relies on the integration of multiple methodological approaches — observational studies, simulations, clinical trials (when feasible), and case reports —, each designed to address specific research questions. In this regard, we do not believe that there is yet a sufficiently broad and consistent body of evidence to support the clinical effectiveness of LifeVac® in real-world FBAO cases. Our study, like others based on simulation, is intended to contribute just one more piece to this complex scientific puzzle. The interpretation that the currently published data is insufficient to establish a well-founded judgment, with robust evidence, is not a personal impression. It is a finding made by other researchers interested in the subject, which has also contributed to the growing attention of other research groups.^1^^,^^5^

Another relevant observation from our experiment was the variability in pressure levels generated by different operators. We believe this variation reflects individual differences in biomechanical efficiency, which is common in manual procedures. We hypothesize that this variability could be minimized through systematic training and standardized protocols for device use. Nevertheless, we cannot rule out the possibility of intrinsic limitations of the device itself when operated by different individuals. Although untrained individuals can use these anti-asphyxia devices with some effectiveness, proper training on the protocol to be recommended in the management of FBAO is crucial for the best management of the clinical scenario.^5^^,^^7^

In summary, we appreciate the constructive criticism and agree that simulation-based results should be interpreted with caution. Nonetheless, we believe that the results of our study – by providing objective pressure data – contribute to the advancement of knowledge about alternative interventions for FBAO since they are presented with due methodological transparency and a clear discussion of their limitations.

Once again, we would like to thank you for the opportunity to discuss such a relevant topic and for helping to improve the critical interpretation of the results presented in our manuscript.

## Conflicts of interest

The authors declare no conflicts of interest.
